# Numerical Forecast Correction of Temperature and Wind Using a Single-Station Single-Time Spatial LightGBM Method

**DOI:** 10.3390/s22010193

**Published:** 2021-12-28

**Authors:** Rongnian Tang, Yuke Ning, Chuang Li, Wen Feng, Youlong Chen, Xiaofeng Xie

**Affiliations:** 1 Electrical and Mechanical College, Hainan University, Haikou 570228, China; rn.tang@hainanu.edu.cn (R.T.); 19080200210007@hainanu.edu.cn (Y.N.); lc@hainanu.edu.cn (C.L.); 2 Hainan Meteorological Observatory, Haikou 570203, China; fengwen202112@163.com (W.F.); chenyoulong2021@163.com (Y.C.); 3 Key Laboratory of South China Sea Meteorological Disaster Prevention and Mitigation of Hainan Province, Haikou 570203, China

**Keywords:** temperature, wind, forecast, spatial feature, LightGBM, weather correction

## Abstract

Achieving high-performance numerical weather prediction (NWP) is important for people’s livelihoods and for socioeconomic development. However, NWP is obtained by solving differential equations with globally observed data without capturing enough local and spatial information at the observed station. To improve the forecasting performance, we propose a novel spatial lightGBM (Light Gradient Boosting Machine) model to correct the numerical forecast results at each observation station. By capturing the local spatial information of stations and using a single-station single-time strategy, the proposed method can incorporate the observed data and model data to achieve high-performance correction of medium-range predictions. Experimental results for temperature and wind prediction in Hainan Province show that the proposed correction method performs well compared with the ECWMF model and outperforms other competing methods.

## 1. Introduction

Numerical weather prediction (NWP), which can provide high-performance weather prediction for the prevention and mitigation of weather-related disasters, is one of the main objective forecast tools available for scientific research and operational forecasting. However, because NWP models cannot fully simulate the real atmosphere, we need to design effective correction methods to modify their results [1,2,3,4,5,6,7]. There are two types of methods in this regard: traditional statistical modeling methods and machine learning-based methods.

Traditional statistical modeling methods correct the error of the dynamic equations of atmospheric motion with a statistical tool, and many studies have been conducted in which this approach to correction has been implemented. For instance, Howard et al. [8] proposed a simple and computationally inexpensive method based on the linear theory of neutral boundary-layer flow over hills to recover a realistic wind profile in the lower boundary layer from NWP and provide an approximate correction for local topography. Verspeek et al. [9] proposed a method for estimating correction tables based on NWP ocean calibration residuals. Dong et al. [10] proposed a linear correction method based on the wavelet transform, and the low-frequency stationary NWP wind speed obtained by wavelet multi-resolution analysis was corrected by this linear correction method. Auligné et al. [11] proposed a variational bias correction scheme to separate the observation bias from the systematic errors in the background field of the observed data in order to prevent the analysis from drifting towards its own climate. However, these traditional statistical modeling methods are prone to suffering from the problem of empiricism, which means the model is designed based on the relationship between variables and the hypothesized distribution of observations.

With the improvement of computing power, many machine learning-based methods have been widely proposed to correct forecast deviations. Machine learning is a data-driven model that can automatically learn the relationship between the observation and prediction from a large amount of historical data. This type of method completely relies on the historical dataset and is highly robust. Wang et al. [12] proposed an end-to-end deep learning algorithm to correctly forecast the weather, and a novel negative log-likelihood error loss function was constructed to learn the network weight. Lauret et al. [13] proposed the use of artificial neural networks as a post-processing technique in order to improve mesoscale WRF solar radiation outputs. Liu et al. [14] proposed three NWP correction methods based on multiple linear regression, a radial basis function neural network, and an Elman neural network. Buhan et al. [15] proposed the use of an artificial neural network and a support vector machine model to learn the relationship between the wind patterns of NWP data and reference wind mast measurements. These methods involve the design of a single regression/classification learner to establish the relationship between the data and label, avoiding the need to deal with the complex correlation between the meteorological variables in the weather data.

To alleviate the systematic bias from one single learner, the Random Forest method has been commonly used for weather forecast correction. This method can generate multiple weakly-supervised decision trees and combine them by a certain strategy to achieve high performance. It can fully mine the important information of complex elements. McCandless et al. [16] used the Random Forest method to generate the cloud mask from GOES−16 radiances to correctly predict solar irradiance reaching the Earth’s surface for more accurate solar power forecasting. Buhan et al. [15] used the AdaBoost (adaptive boosting) algorithm to combine NWP data, thus providing a proper combination of meteorological grid data from a set of surrounding grids. Du et al. [17] proposed an ensemble method to forecast wind power production, which was created by blending the results derived from three algorithms through a Bayesian model average. Kang et al. [18] combined the support vector machine, Random Forest, gradient boosting decision tree (GBDT) and XGBoost methods for predicting weather at a lead time of 10 days with a 1 km spatial resolution 1 h temporal resolution.

In this paper, by considering the performance and computational demands, a lightweight Random Forest-based method—namely, “single-station single-time spatial LightGBM”—is proposed to achieve weather forecast correction. Compared with the traditional lightGBM model, our proposed method can capture the local spatial information of observed stations and apply the single-station single-time training strategy in the learning of the correction’s model. It is a further extension of the lightGBM model based on the fact that the weather has local similarity characteristics in the local area.

The main contributions of this paper are threefold:

(1) A novel spatial lightGBM method is proposed to capture local spatial information of observation stations for improved correction performance.
(2) A single-station single-time training strategy is used for spatial lightGBM model learning. This strategy can reflect the differences in the geographic location of different stations because of decoupling the spatiotemporal correlation of stations.
(3) The mechanism of the single-station single-time training strategy is explained by calculating the importance of each meteorological element to the temperature prediction of the spatial lightGBM. Our strategy can reflect the differences of different stations due to topographic effects.

The remainder of the paper is organized as follows: In Section 2, the selected experimental data and sample construction method are described in detail. In Section 3, we present the single-station single-time spatial lightGBM method. Extensive experimental results and related discussion are provided in Section 4 to demonstrate the effectiveness of the proposed method. Lastly, some conclusions are provided in Section 5.

## 2. Data and Sample Construction

In order to test the proposed method in practical terms, we take the 18 observation stations in Hainan Province as experimental objects to correct the prediction of temperature and wind from the ECWMF model. The study of temperature and wind is critical for the development of the island climate, which has been one of the hot researched areas in the weather forecast. For instance, Devi R M et al. [19] use multivariate regression and autoregression to investigate the change in land surface temperatures on the island from 2000 to 2019. Nezhad M M et al. [20] proposed a method for assessing and mapping the wind energy potential of near- and off-shore areas by means of multisensor satellites and applied it to a case study area on the northwest coast of Sicily. Kim S. [21] create a model for the temperature forecast of an island community by using multilinear regression to take into account multiple variables. Darmiyati Muksin et al. [22] analyze the effect of ENSO (El Nino Southern Oscillation) on climate parameters (rainfall and air temperature) that occur on Morotai Island. In this paper, supported by the local meteorological department, we mainly focus on the correction of temperature and wind forecast in Hainan Island. The model data and observed data of the 18 cities are introduced as follows.

### 2.1. Model Data

**Figure 1:**
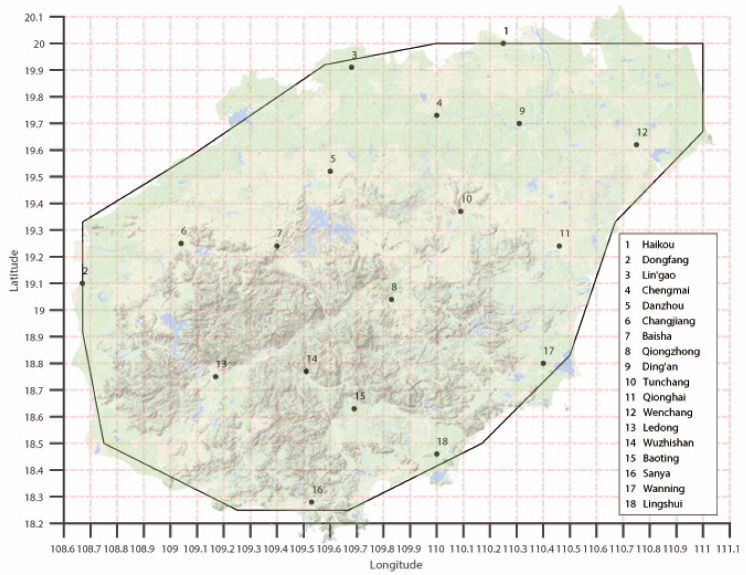
Distribution of the 18 observation stations in Hainan Province.

The three-hourly forecast data of the ECMWF model initialized at 0000 and 1200 UTC up to a lead time of 168 h from January 2018 to December 2020 are used in this study. The model data cover the main observation stations of 18 cities in Hainan Province, and the grid-point data corresponding to the latitude and longitude coordinates of the main sites of the 18 cities are obtained through the upper-right corner principle. Figure 1 shows the distribution of the 18 observation stations in Hainan Province.

After referring to the relevant literature, we select 49 predictors based on meteorological intuition from the ECWMF results for the correction of temperature and wind prediction. Table 1 lists these predictors and their abbreviations.

### 2.2. Observation Data

Because of the rich information that the historical observation data contain, we also use observation data from the observation stations of the 18 cities in Hainan Province to correct the ECMWF prediction. Taking into account the need for temperature and wind correction, we choose eight observational elements from each station. Table 2 lists these observational elements and their abbreviations.

### 2.3. Sample Construction

**Figure 2:**
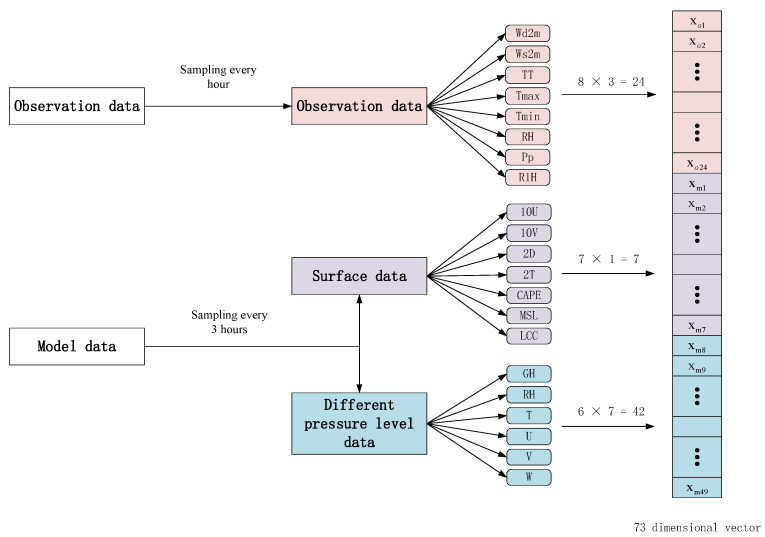
Schematic illustration of the sample construction process.

In machine learning, the data need to be constructed into samples for model learning. In this paper, we construct a sample every 3 h, which includes 73 features and labels. The formula for one station can be expressed as follows:

(1) $$S=\{x_{mi},x_{m2},\cdots ,x_{m49},x_{o1},x_{o2},\cdots ,x_{o24},y_{label}\}$$

where $x_{m1}-x_{m49}$ represents the 49 predictors from the ECWMF results, $x_{o1}-x_{o24}$ is derived from the 3 h observation data (eight observation elements per hour), and $y_{label}$ is the real temperature or wind. Figure 2 illustrates the sample construction process.

## 3. Method

### 3.1. LightGBM Model

The LightGBM [23] model is a state-of-the-art boosting method for fitting the relationship between features and labels. Its purpose is to optimize the efficiency and scalability on the basis of the GBDT algorithm [24]. The GBDT model uses the negative gradient of the loss function to learn the base learner and obtain the final strong learner through weighted summation. In GBDT, the information gain is usually measured by the variance after splitting:

(2) $$V_{j|0}(d)=\frac{1}{n_{o}}(\frac{{\left(\sum_{\left\{x_{i}\in O:X_{ij}\leq d\right\}}g_{i}\right)}^{2}}{n_{l|0}^{j}(d)}+\frac{{\left(\sum_{\left\{x_{i}\in O:X_{ij}>d\right\}}g_{i}\right)}^{2}}{n_{r|0}^{j}(d)})$$

where *O* is the training dataset on a fixed node of the decision tree, and $n_{o}=\sum^{\;}I\left[x_{i}\in O\right]$, $n_{l|0}^{j}\left(d\right)=\sum^{\;}I\left[x_{i}\in O:x_{ij}\leq d\right]$, $n_{r|0}^{j}\left(d\right)=\sum^{\;}I[x_{i}\in O:x_{ij}>d]$. For feature *j*, each decision tree selects $d_{j}^{*}={\mathrm{argmax}}_{d}V_{j}\left(d\right)$ and divides the data into two left and right sub-nodes according to the value of feature *j* at $d_{j}^{*}$. However, GBDT has low efficiency and high computational cost in regression since it needs to traverse the samples and features of each node to find the best splitting point in each iteration.

**Figure 3:**
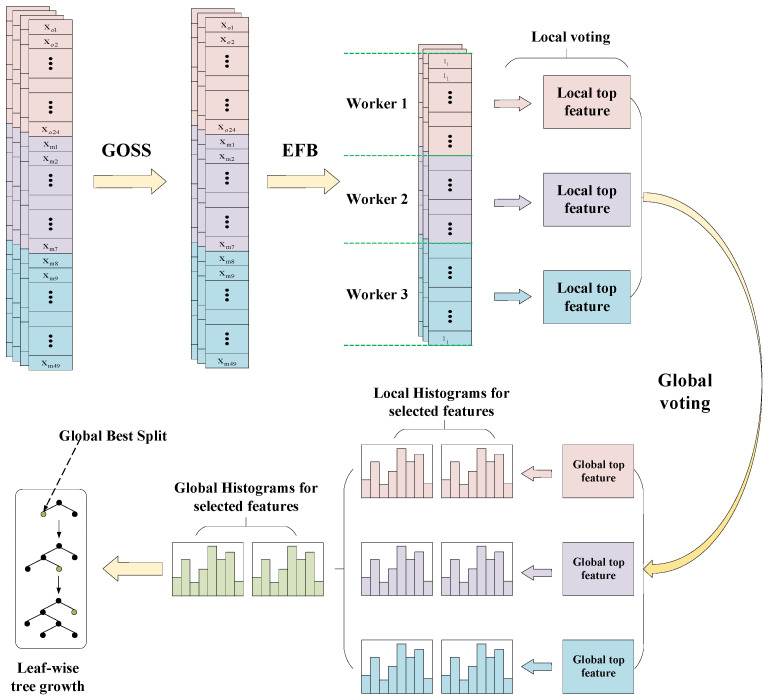
Schematic illustration of the lightGBM model.

LightGBM was further developed to address the computational cost problem by designing a gradient-based one-side sampling (GOSS) and exclusive feature binding (EFB) operator. Specifically, GOSS down-samples the sample instances and randomly discards those instances with small gradients, while EFB can reduce the complexity of the feature space by binding the mutually exclusive features. The information gain of the sample was modified as follows:

(3) $${\tilde{V}}_{j}(d)=\frac{1}{n_{o}}(\frac{{\left(\sum_{x_{i}\in A_{l}}g_{i}+\frac{1-a}{b}\sum_{x_{i}\in B_{l}}g_{i}\right)}^{2}}{n_{l}^{j}(d)}+\frac{{\left(\sum_{x_{i}\in A_{r}}g_{i}+\frac{1-a}{b}\sum_{x_{i}\in B_{r}}g_{i}\right)}^{2}}{n_{r}^{j}(d)})$$

where $A_{l}=\left\{x_{i}\in B:x_{ij}\leq d\right\}$, $A_{r}=\{x_{i}\in A:x_{ij}>d\}$ and $B_{l}=\left\{x_{i}\in B:x_{ij}\leq d\right\}$, $B_{r}=\{x_{i}\in B:x_{ij}>d\}$. Subset A is the first a × 100% sample with the larger gradient, and subset B is the remaining (1 − a) × 100% samples with small gradients. In addition, LightGBM also provides histograms to select features and employs a leaf-wise growth strategy to reduce the growth of leaf nodes. The framework of the lightGBM model is shown in Figure 3.

### 3.2. The Single-Station Single-Time Spatial LightGBM

The lightGBM model can alleviate the computational cost to achieve high performance. However, lightGBM cannot take into account the local spatial information of the sample, which plays an important role in weather forecasting. To this end, we extended the lightGBM to a single-station single-time spatial lightGBM. It consists of spatial sample construction and a single-station single-time training strategy.

For spatial sample construction, in order to embed more spatial information into samples, the two nearest stations’ observed data were added into samples. Additionally, the selection of the two nearest stations is based on the straight-line distance of the longitude and latitude coordinates of the stations. The spatial sample can be expressed as

(4) $$S=\{x_{mi},x_{m2},\cdots ,x_{m49},x_{o1},x_{o2},\cdots ,x_{o24},{\overset{⌢}{x}}_{o1},{\overset{⌢}{x}}_{o2},\cdots ,{\overset{⌢}{x}}_{o48},y_{label}\}$$

where ${\overset{⌢}{x}}_{o1-o48}$ denotes the 3-h observation data from the two nearest stations.

**Figure 4:**
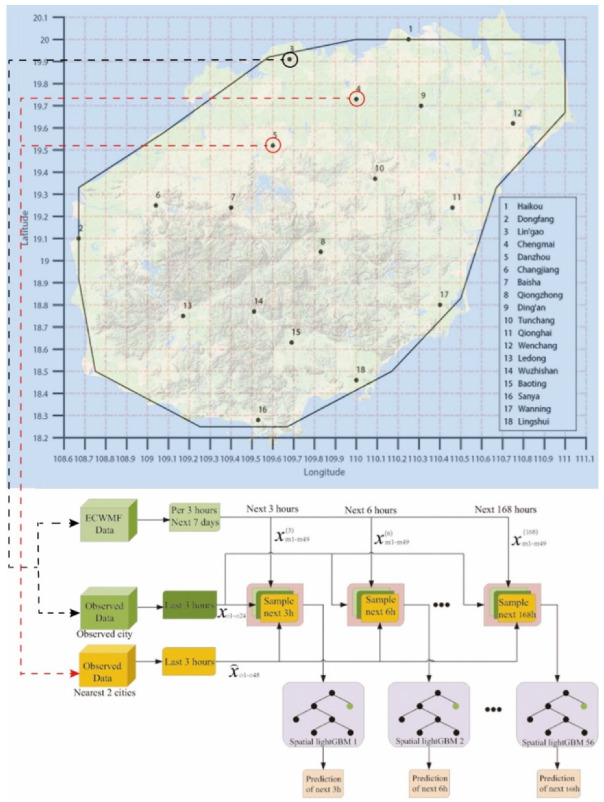
The single-station single-time spatial lightGBM for Lingao city.

For the training strategy, we designed a single-station single-time strategy to train the spatial lightGBM model in 18 cities separately. This strategy can decouple the impact of geographical or climate differences contained in different geographical locations of the 18 cities in Hainan Province on the model. More specifically, we forecast the weather for the next 7 days at 3 h intervals for each city. Additionally, we need to learn 56 spatial lightGBM models for each city. Figure 4 illustrates the single-station single-time spatial lightGBM.

## 4. Results and Discussion

### 4.1. Evaluation Index

In order to evaluate the performance of the proposed method in terms of temperature and wind correction, three evaluation indexes [the root-mean-square error (RMSE), accuracy rate of less than 2 °C and 1 °C, the wind speed classification forecast accuracy rate] are employed in the analysis of the experimental results. We briefly introduce these indexes as follows:

1. The RMSE is defined as:
  (5) $$RMSE=\sqrt{\frac{\sum_{i=1}^{N}{\left(x_{i}-x\right)}^{2}}{N}}$$
  where $x_{i}$ is the forecast value, *x* is the true value, and *N* is the total number.

2. The accuracy rate of less than 2 °C and 1 °C is defined as follows:
  (6) $$TT_{k}=\frac{N_{r}}{N}$$
  where *N* represents the total number of forecasts, and $N_{r}$ represents the number of correct forecasts. When $N_{r}$ represents the number of times the error between the predicted value and the true value is within 1 °C, *k* = 1, and at this time, $TT_{1}$ represents the accuracy rate of <1 °C. Likewise, when $N_{r}$ represents the number of times the error between the predicted value and the true value is within 2 °C, *k* = 2, and at this time, $TT_{2}$ represents the accuracy rate of <2 °C.

3. The wind speed classification forecast accuracy rate is defined as follows:
  (7) $$AC_{s}=\frac{\sum_{i=1}^{k}NR_{si}}{NF}$$
  where $NR_{si}$ is the correct number of wind forecasts for the *i*th level, indicating that the forecast wind speed and the actual wind speed are at the same level; NF is the total number of forecasts; and *k* is the wind speed forecast level, of which there are 13.

### 4.2. Experimental Results and Discussion

In this section, we analyze and discuss the performance of the proposed method. We begin by comparing the ECWMF model and the proposed method in terms of their temperature and wind forecasts to prove the effectiveness of the correction. Then, we further compare the proposed correction method with other competing correction methods before lastly discussing the benefits of the single-station single-time strategy.

#### 4.2.1. Correction Performance Analysis

**Figure 5:**
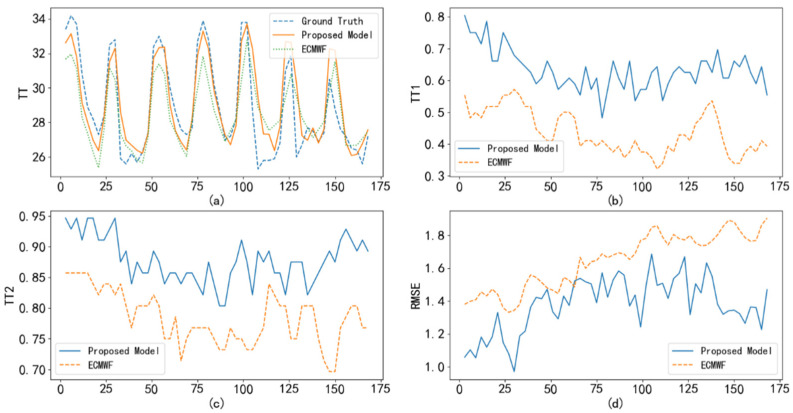
Comparison of the ECWMF model and the proposed method in terms of their temperature forecast at Haikou Station: (**a**) real temperature versus ECWMF prediction and proposed correction; (**b**) $TT_{1}$ index comparison; (**c**) $TT_{2}$ index comparison; (**d**) RMSE index comparison.

**Figure 6:**
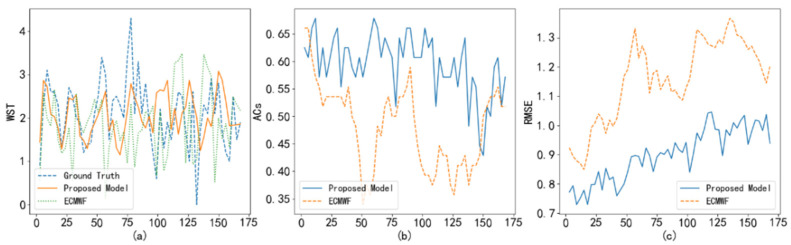
Comparison of the ECWMF model and the proposed method in terms of their wind forecast at Haikou Station: (**a**) real wind speed versus ECWMF prediction and proposed correction; (**b**) $AC_{s}$ index comparison; (**c**) RMSE index comparison.

To test the correction performance of the proposed method, we compare the temperature and wind predictions of the ECWMF model with the corresponding corrections learned by the proposed method. Taking Haikou station on 6 September 2020 as an example, Figure 5a shows the real temperature versus ECWMF prediction and proposed correction. It can be seen that the correction results are closer to the real temperature than the ECWMF model results. Furthermore, the $TT_{1}$, $TT_{2}$, and RMSE index comparisons in Figure 5b–d also support the above inference. On the other hand, we also show the prediction and correction of wind speed in Figure 6. As shown in Figure 6, the correction of wind speed learned by the proposed method outperforms the ECWMF prediction.

**Figure 7:**
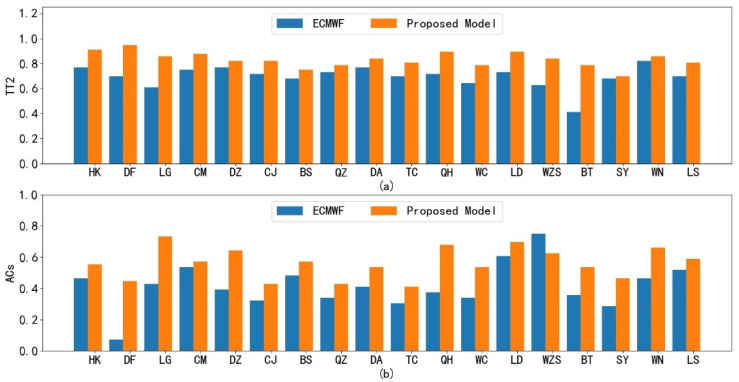
The comparisons of the ECWMF model and the proposed method at 18 cities: (**a**) average $TT_{2}$ index of temperature forecast for ECWMF prediction and proposed correction; (**b**) $AC_{s}$ index of wind speed for ECWMF prediction and proposed correction.

**Figure 8:**
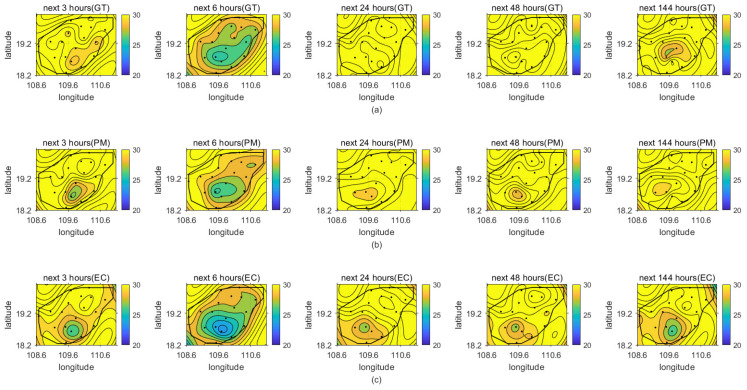
Temperature fields of 18 cities for the next 3, 6, 24, 48, and 144 h: (**a**) real temperature; (**b**) proposed correction; (**c**) ECWMF prediction.

After proving the superior performance with respect to Haikou station, we further show the correction results for the 18 cities of Hainan Province. We provide a 7-day forecast with 3 h intervals at a start time of 0800 UTC 6 September 2020. Figure 7 shows the 7-day average $TT_{2}$ index of the ECWMF model and the proposed method at the 18 cities, separately. From Figure 7, it can be seen that the proposed method achieves higher performance in its 7-day forecast than the ECWMF model. To more intuitively illustrate this and provide more detail, we select some predictions at specific times from the 7-day temperature forecast. Figure 8 shows the temperature field of the real temperature versus the ECWMF prediction and the proposed correction at the 18 cities for the next 3, 6, 24, 48, and 144 h, separately. We can see from the results that the proposed spatial lightGBM model can correct the ECWMF prediction at each station to ensure that the predicted temperature field can press closer to the real situation, supporting the high average performance of the proposed method illustrated in Figure 7.

#### 4.2.2. Comparison with Competing Correction Methods

**Figure 9:**
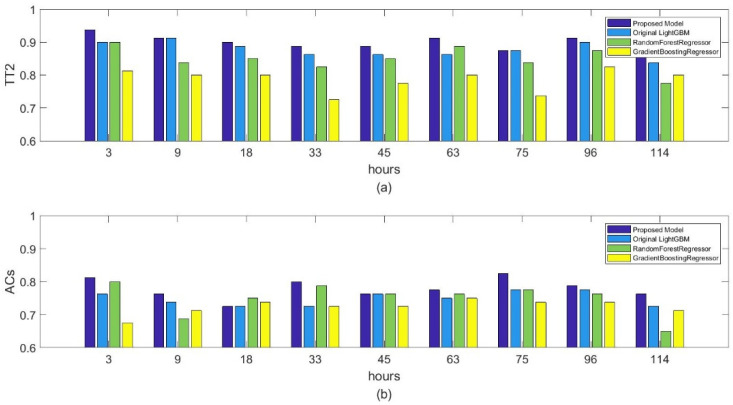
Comparison of various correction methods in the next 3, 9, 18, 33, 45, 63, 75, 96, and 114 h: (**a**) $TT_{2}$ index comparison; (**b**) $AC_{s}$ index comparison.

To fairly evaluate the correction performance of the proposed method, we also carry out an experiment to compare the proposed method with other competing correction methods, namely Random Forest, GBDT, and the original lightGBM. Figure 9 shows the correction performance of temperature and wind for all the methods. As we can see, the proposed spatial lightGBM performs better than Random Forest, GBDT, and the original lightGBM. Compared to other competing methods, the proposed method can capture the local spatial information by using the two nearest stations’ observed data and train the spatial lightGBM model in 18 cities separately to alleviate the impact of geographical or climate differences contained in different geographical locations of the 18 cities. This is particularly evident in the comparison between the original lightGBM and the spatial lightGBM. Therefore, the high correction performance of the proposed method might be attributable in part to the ability of the sample to capture local spatial information and training strategy.

In addition, we also compare the computational loads of the proposed method with Random Forest and GBDT to demonstrate the efficiency of lightGBM. As shown in Table 3, the proposed spatial lightGBM method requires less training time than Random Forest and GBDT because of the gradient-based one-sided sampling and exclusive feature binding operator in lightGBM. The results in Figure 9 and Table 3 indicate that the proposed spatial lightGBM method can achieve a better trade-off between correction performance and efficiency. The proposed method could be applied in large areas with a large number of meteorological stations because the spatial lightGBM method requires less training time and lower computational loads. On the other hand, the proposed method will not be easily affected by the few meteorological stations because the single-station single-time training strategy can keep the independence of stations.

#### 4.2.3. Advantages of the Single-Station Single-Time Strategy

**Figure 10:**
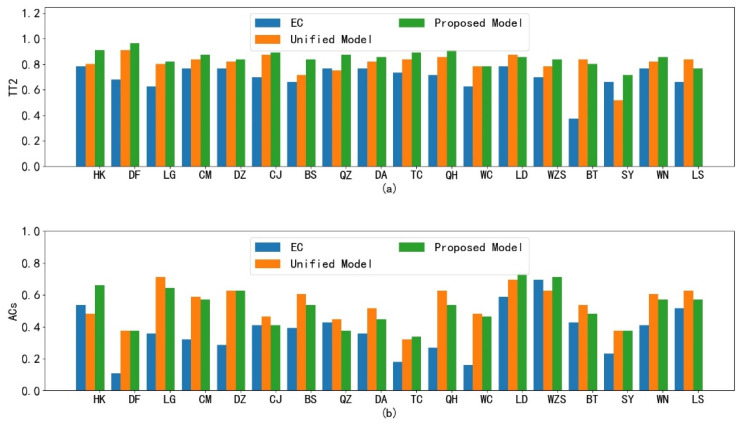
Comparison between the proposed spatial lightGBM and unified spatial lightGBM in terms of their temperature and wind forecasts: (**a**) $TT_{2}$ index comparison; (**b**) $AC_{s}$ index comparison.

Lastly, we discuss the advantages of the single-station single-time strategy. In our study, we employ this strategy to construct 56 × 18 spatial lightGBM models for 7-day forecasts at 18 cities. Additionally, in contrast, we train 56 unified spatial lightGBM models from 7-day forecasts by using all 18 cities for ease of comparison, without distinguishing between stations and forecast times. Figure 10 compares the multiple spatial lightGBM models and the unified one in terms of their temperature and wind forecasts. It can be seen that our single-station single-time strategy can improve the correction performance compared with the unified model. At most stations, the proposed method outperforms the unified model, which helps prove the effectiveness of the single-station single-time strategy.

**Figure 11:**
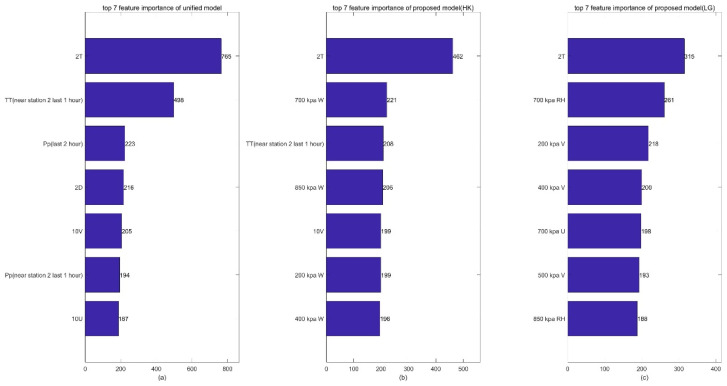
Top 7 features in terms of their importance to the temperature prediction calculated by the proposed spatial lightGBM and the unified spatial lightGBM: (**a**) top 7 feature importance of unified model; (**b**) top 7 feature importance of proposed model in Haikou; (**c**) top 7 feature importance of proposed model in Lingao.

Furthermore, to explain the effectiveness of the single-station single-time strategy, we calculate the times of each feature to split the temperature prediction from the proposed spatial lightGBM and unified spatial lightGBM, which is denoted by feature importance. Taking Haikou and Lingao city as examples, Figure 11 shows the top seven features in terms of their importance as calculated by the two lightGBM models. It can be seen that the unified model has the same order of importance for Haikou and Lingao; however, the proposed model obtains a different order for Haikou and Lingao. Specifically, the top seven features of the unified model are 2 T, TT, Pp, 2 D, 10 V, Pp (near the station), and10 U for Haikou and Lingao. However, in the proposed model, the top five features at Haikou are 2 T, 700 kapW, TT, 850 kapW, 10 V, 200 kpaw, 400 kpa, while at Lingao, they are 2 T, 700 kpaRH, 200 kpaV, 400 kpaV, 700 kpaU, 500 kpaV, and 850 kpaRH. This indicates that the proposed single-station single-time strategy can reflect the differences of different stations due to topographic effects, but the unified model fails in this regard. The main contributions of the proposed method are spatial information extraction and training strategy. In particular, some related studies also employed similar processing. For instance, Zhong J et al. [25] used a novel feature engineering approach to incorporate spatial effects from meteorological data for PM2.5 prediction. Haochen L I. et al. [3] take surrounding spatial points of the Beijing area into account in a unified way, which performs better when the forecast time is longer. Mousavi S M et al. [26] present a deep learning method for the single-station earthquake location by using two separate Bayesian neural networks. However, it is firstly used in temperature and wind forecast correction.

## 5. Conclusions

In this paper, by capturing the local spatial information of stations and using a single-station single-time strategy, we design a novel and robust lightGBM model to correct temperature and wind NWP forecasts. For the correction performance evaluation, the $TT_{1}$, $TT_{2}$, $AC_{s}$, and RMSE index of the proposed method outperforms the ECWMF prediction, respectively. This proves the proposed method’s high performance for temperature and wind correction. In addition, we also compared the proposed method with the state-of-art method, e.g., Random Forest, GBDT, and the original lightGBM, on the correction performance. The higher accuracies and lower computational time of the proposed method prove its effectiveness. Furthermore, we analyze the importance of features to demonstrate that the proposed method can measure the differences of different stations due to topographic effects, but the unified model fails in this regard. The proposed method can also be applied in the prediction of many other meteorological elements.

## Figures and Tables

**Table 1:** The selected predictors and their abbreviations.

Data Types	Predictors	Abbreviations
Predictors at the surface	10-m *U*-wind component	10 U
	10-m *V*-wind component	10 V
	2-m dewpoint temperature	2 D
	2-m temperature	2 T
	Convective available potential energy	CAPE
	Mean sea level pressure	MSL
	Low cloud cover	LCC
Predictors at different pressure levels (200 kpa, 400 kpa, 500 kpa, 700 kpa, 850 kpa, 925 kpa, 950 kpa)	Geopotential height	GH
	Relative humidity	RH
	Temperature	T
	U component of wind	U
	V component of wind	V
	Vertical velocity	W
	Geopotential height	GH

**Table 2:** The selected observation elements and their abbreviations.

Element	Abbreviation	Element	Abbreviation
2-m wind direction	Wd2 m	Minimum temperature	Tmin
2-m wind speed	Ws2 m	Relative humidity	RH
Temperature	TT	Station pressure	Pp
Maximum temperature	Tmax	Hourly precipitation	R1 H

**Table 3:** Comparison of various correction methods in training time.

	Proposed Model	Original LightGBM	Random Forest	GBDT
Cost Time (s)	19.0	8.5	1772.5	605.3

## Data Availability

Data are available on request due to restrictions, e.g., privacy or ethics. The data presented in this study are available on request from the corresponding author. The data are not publicly available due to confidentiality agreements.
